# Formation of the Heart: Defining Cardiomyocyte Progenitors at Single-Cell Resolution

**DOI:** 10.1007/s11886-023-01880-z

**Published:** 2023-04-29

**Authors:** Richard C. V. Tyser

**Affiliations:** Wellcome-MRC Cambridge Stem Cell Institute, University of Cambridge, Jeffrey Cheah Biomedical Centre, Cambridge, CB2 0AW UK

**Keywords:** Heart development, Cardiac progenitor, Heart field, Myocyte lineage, Cardiomyocyte differentiation

## Abstract

**Purpose of Review:**

Formation of the heart requires the coordinated addition of multiple progenitor sources which have undergone different pathways of specification and differentiation. In this review, I aim to put into context how recent studies defining cardiac progenitor heterogeneity build on our understanding of early heart development and also discuss the questions raised by this new insight.

**Recent Findings:**

With the development of sequencing technologies and imaging approaches, it has been possible to define, at high temporal resolution, the molecular profile and anatomical location of cardiac progenitors at the single-cell level, during the formation of the mammalian heart.

**Summary:**

Given the recent progress in our understanding of early heart development and technical advances in high-resolution time-lapse imaging and lineage analysis, we are now in a position of great potential, allowing us to resolve heart formation at previously impossible levels of detail. Understanding how this essential organ forms not only addresses questions of fundamental biological significance but also provides a blueprint for strategies to both treat and model heart disease.

## Background and Introduction

The heart, blood, and vasculature are the first functional organ system to form during embryogenesis, essential in providing the developing embryo with sufficient oxygen and nutrients [1, 2]. Decisions to commit to a cardiac cell fate are therefore taken early in development, and perturbations to molecular and morphological events of this process frequently result in congenital defects [3].

**Fig. 1:**
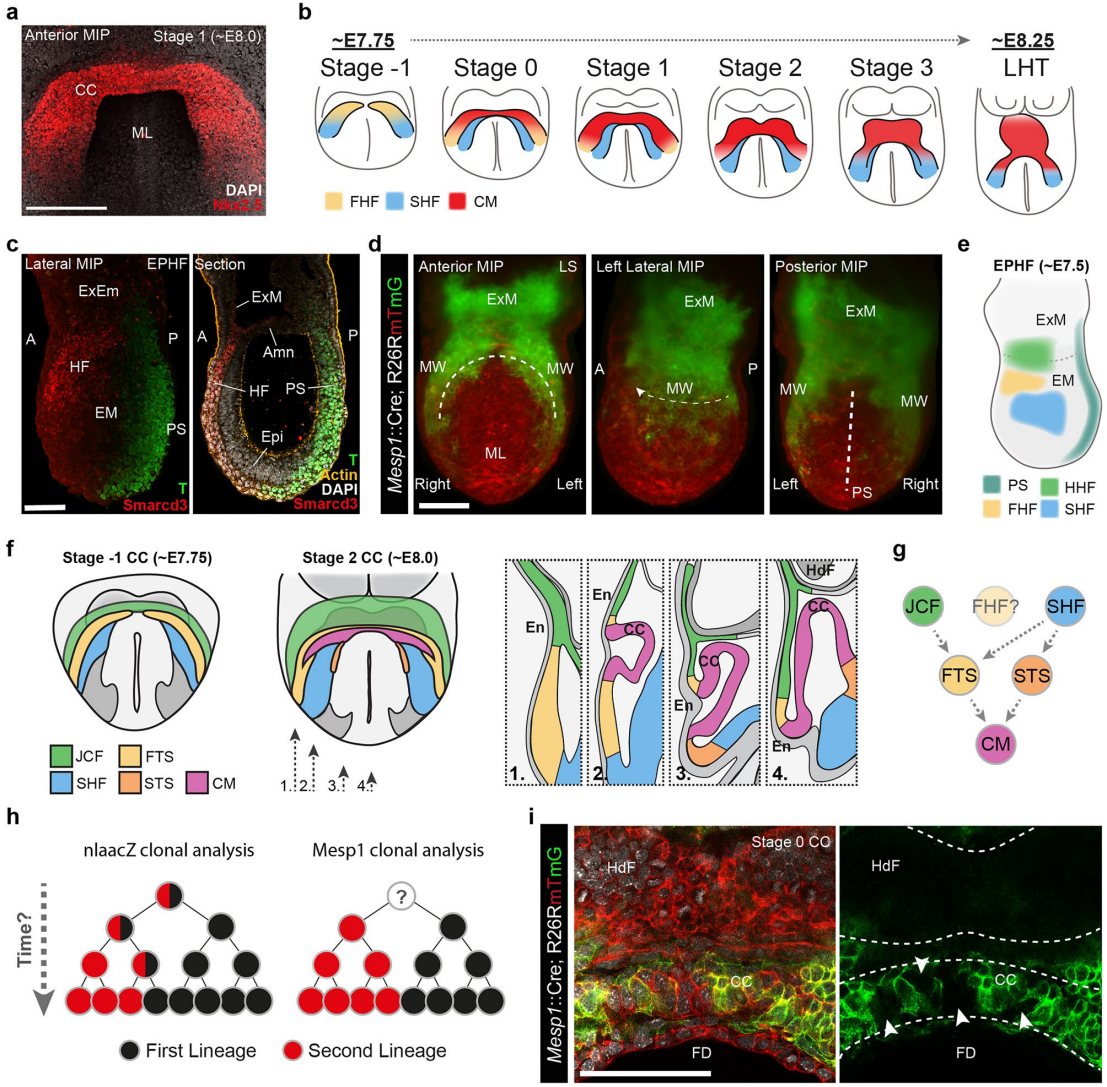
**Defining Cardiomyocyte Progenitors at Single Cell Resolution.** **a**, Anterior maximum intensity projection (MIP) of a Stage 1 cardiac crescent showing expression of Nkx2-5 and DAPI using whole mount immunohistochemistry. Scale bar, 200 μm, CC; Cardiac Crescent, ML; Midline. **b**, Schematic highlighting the stages of cardiac crescent development and the classic view of hear fields. FHF; First Heart Field, SHF; Second Heart Field, CM; Cardiomyocyte, LHT; Linear Heart Tube. **c**, Lateral MIP (left panel) and medial section (right panel) of an early pre-headfold (EPHF) stage embryo (EPHF) highlighting the primitive Streak as marked by Brachyury (T) and anterior Smarcd3 positive heart field. Scare bar, 100 μm. PS; Primitive Streak, MW; Mesodermal Wing, ExM; Extraembryonic Mesoderm, A; Anterior, P; Posterior, HF; Heart Field, ExEm; Extraembryonic, Em; Embryonic, Epi; Epiblast, Amn; Amnion. **d**, MIP of a late streak (LS) stage (~E7.25) embryo from different angles using light sheet microscopy. Mesp1-Cre labelling of mesoderm (green) highlights the bilateral mesodermal wings after they have left the primitive streak. Cell membranes labelled in red, Scale bar, 100 μm. **e**, Schematic of early pre-headfold (EPHF) stage embryo depicting traditional heart fields as well as the recently defined Hand1 positive cardiac progenitors (HHF, Zhang et al. [23••]). Dotted line represents extraembryonic/embryonic boundary. **f**, Schematic highlighting the anatomical location of cardiac progenitor transcriptional states in the anterior region of cardiac crescent Stage-1 and Stage 2 embryos. As identified in Tyser et al. [22••]. Arrows represent the location of sagittal sections in dotted boxes. JCF; Juxta Cardiac Field, FTS; FHF Transition State, STS; SHF Transition State, En; Endoderm, HdF; Headfold. **g**, Schematic diagram summarizing the trajectories linking the cardiac progenitor states depicted in panel **f**. Due to the timepoints examined we could not determine whether a FHF population exists that is transcriptionally distinct from the FHF transition state. **h**, Schematic diagrams representing previous clonal analysis experiments highlighting the difference between approaches. **i**, Frontal section through a stage 1 embryo highlighting cardiomyocyte in the cardiac crescent not labelled using *Mesp1*-Cre (arrow heads). Cell membranes labelled in red, *Mesp1*-Cre labelled cells in green. Scale bar, 100 μm. FD; Foregut Diverticulum. (Panels **a**, **b**, **f**, **g** from [22••]. Reprinted with permission from AAAS)

The first morphologically recognizable heart structure in the developing embryo is the cardiac crescent, which forms at around day 20 of gestation in humans (embryonic day (E) 8.0 in mice, Fig. 1a) [4]. The cardiac crescent is an arc of immature cardiomyocytes in the anterior of the embryo and is where contraction first initiates [5, 6]. Formation of the cardiac crescent requires the coordinated addition of multiple progenitor sources, which have undergone different pathways of specification and differentiation [7, 8]. In vertebrates, this process occurs when stem cells of the epiblast undergo gastrulation, the fundamental process by which the body plan is laid down [9, 10]. During this process, nascent mesoderm forms as pluripotent cells of the epiblast undergo an epithelial to mesenchymal transition and ingress through the primitive streak. This occurs in the caudal region of the embryo at around day 14 in the human (~ E6.25 mouse) [11]. The nascent mesoderm then migrates as two bilateral sheets (mesodermal wings) toward the anterior of the embryo (Fig. 1b, c). In this anterior region, the mesodermal wings meet at the midline and buckle to form the cardiac crescent [12]. During this migration, the nascent mesoderm specifies to form cardiac progenitors before differentiating into cell types such as immature cardiomyocytes. The cardiac crescent subsequently fuses at the midline to create the linear heart tube, before undergoing a complex process of morphogenetic remodeling to form the 4-chambered heart [13]. During these later stages of development, a heterogeneous population of progenitors continue to add to the heart, differentiating into a diverse range of cell types and enabling the heart to grow and maintain its vital function [14–16].

Understanding heart formation aids our knowledge of congenital heart disease, allowing us to establish the underlying causes of defects. Such understanding provides a blueprint to generate biology relevant in vitro models as well as insight into regenerative approaches to treat heart diseases, such as the damage caused during a heart attack. Congenital heart defects are the most common type of birth defect, being diagnosed in at least 1 in 125 births: equating to around 98 babies each day in the European Union [17]. In humans, the developing heart is most vulnerable to defects between 3 and 7 weeks, coinciding with when heart formation first begins and when the largest morphological rearrangements occur [18, 19]. Heart attacks, the manifestation of coronary artery disease, are the leading cause of death worldwide, accounting for 1.8 million deaths each year in Europe alone [20, 21]. The development of regenerative approaches to treat this disease by the restoration of function is vital.

Recently, single-cell-based approaches have revealed an unappreciated complexity in regards to cardiac progenitor heterogeneity, providing new and exciting opportunities to learn how the heart forms during early embryonic development [22••, 23••, 24–28]. This review will examine how our recent transcriptional and anatomical characterization of cardiac progenitors relates to previously defined heart fields and myocyte lineages as well as explore the questions this characterization raises.

## Heart Fields

During formation of the heart, cardiac progenitors reside in bilateral regions of the embryo, termed heart fields (Fig. 1b). These heart-forming fields are anatomically defined based on the expression patterns of molecular markers (Table 1). Classically, cardiac progenitors have been attributed to two main heart fields, termed the first and second heart fields (FHF and SHF, Fig. 1b) [29]. The FHF represents cardiac progenitors which rapidly differentiate to give rise to the cardiomyocytes of the cardiac crescent and lose proliferative capacity. The SHF is a wider domain of progenitors, dorsal and medial to the cardiac crescent, which maintain their proliferative capacity and continue to add cells to the cardiac crescent as it develops [30]. As a result of advances in sequencing technologies and multiplexed imaging approaches, it has been possible to define, at high temporal resolution, the molecular profile of cardiac progenitors at the single-cell level, as well as anatomically map these transcriptionally distinct progenitor states during heart formation in the intact embryo [22••, 23••]. This has enabled a significant advance in our understanding of cardiac progenitors and thus our ability to define heart fields, taking into account a combination of genes rather than single markers.

**Table 1:** Marker gene expression in different cardiac progenitor states as defined by Tyser et al. [22••]

	Cardiac progenitor cell states				
Genes	CM	FHF-TS	JCF	SHF-TS	SHF
*Nkx2.5*	+++	+++	-	+++	++
*Isl1*	-	++	+	+++	++
*Sfrp5*	++	+++	+++	+++	++
*Tnnt2*	+++	+++	+++	+++	++
*Myom1*	+++	+	-	++	-
*Actn2*	+++	-	-	-	-
*Tbx5*	+++	+++	++	-	+
*Hcn4*	++	++	-	-	-
*Hand1*	-	++	+++	-	-
*Smarcd3*	++	++	++	-	-
*Mab21l2*	-	++	++	-	-
*Hoxd1*	-	-	++	-	-
*Tbx1*	-	-	-	++	++
*Fgf10*	-	-	-	++	++
*Hoxb1*	-	-	++	-	++
*Tbx18*	-	-	-	-	-
*Bmp4*	-	++	+++	+	++
*Wnt2*	+++	+++	+++	-	+

Expression level is based on median expression per cluster. Data accessible at https://marionilab.cruk.cam.ac.uk/heartAtlas/

*CM* cardiomyocytes, *FHF-TS* first heart field transition state, *JCF* Juxta Cardiac Field, *SHF-TS* second heart field transition state, *SHF* second heart field

Characterization of a distinct FHF progenitor has remained elusive given its rapid differentiation into cardiomyocytes at the onset of cardiac crescent formation. Thus, there are limited FHF markers due to the difficulty in distinguishing unique genes, which aren’t broad markers of cardiomyocyte differentiation (e.g., *Actn2*, *Tnnt*2) (Table 1). This highlights the difficulty in precisely defining heart fields using markers and means specific contours vary between authors. Defining hearts fields based on marker expression means that the cell populations identified will vary depending on the gene used and the stage of development assessed. Alternatively, defining heart fields based on anatomical location means that heart fields vary depending on the morphological landmark used and the stage of embryonic development (e.g., position within the mesodermal wing or location relative to the cardiac crescent). It also means that classic heart field marker genes overlap with different heart fields (e.g., *Tbx5*, Table 1) [31, 32]. By anatomically characterizing, at high temporal and spatial resolution, progenitor populations based on their entire transcriptome at the single-cell level provide increased clarity when defining heart fields.

Historically, the early cardiomyocytes of the cardiac crescent were termed the FHF; however, given these cells have differentiated into immature cardiomyocytes, they do not truly represent a cardiac progenitor population. In contrast, we recently observed that a FHF-like progenitor state is maintained from emergence of the cardiac crescent to formation of linear heart tube [22••]. Anatomically, this FHF-like state was located at the boundary between more progenitor-like states and differentiating cardiomyocytes (Fig. 1f). This may suggest that FHF progenitors are maintained during cardiac crescent development or that a FHF-like profile represents a transitory molecular state during differentiation toward cardiomyocytes.

SHF cardiac progenitors were initially defined because of their later addition to the heart tube and have been more straightforward to characterize, thanks to their maintained proliferative presence and distinct anatomical location [33–35]. The SHF has been defined based on the expression of genes such as *Fgf10*, *Isl1*, and *Tbx1* and can be subdivided into two anatomically distinct populations termed the posterior SHF (pSHF = Tbx1 positive; Tbx5 positive) and anterior SHF (aSHF = Tbx1 positive; Tbx5 negative) (Table 1) [36, 37]. Based on marker gene expression, aSHF and pSHF progenitors have been detected during early mesoderm formation. When using molecular markers to define heart fields during early development caution is required due to the dynamic and transient nature of gene expression during gastrulation and early organogenesis. For example, the SHF marker, *Islet1*, is broadly expressed in all cardiac progenitors prior to formation of the cardiac crescent but is rapidly downregulated upon cardiomyocyte differentiation over a period of < 6 h (Table 1) [22••, 38, 39]. Thus, it is fundamental to characterize the expression of molecular markers in high temporal resolution during in vivo development, especially when assessing lineage potential using specific marker genes and inducible Cre lines.

Recently, a novel anatomically distinct population of cardiac progenitors located in a region distinct from the traditional FHF and SHFs has been discovered [22••, 23••]. Initially, this population of progenitors resides in a region adjacent to the forming cardiac crescent, at the confluence between the splanchnic and extraembryonic mesoderm (Fig. 1e, f). As the heart begins to form and rostral folding occurs, this population becomes positioned in an arc overlying the cardiac crescent and sandwiched against the endoderm, before extending caudally to also occupy a region at the inflow of the heart (Fig. 1f). As well as being anatomically distinct, this population has a unique transcriptional signature in comparison to previously reported cardiac progenitor populations. It strongly expresses a distinct combination of FHF and SHF markers (e.g., *Tbx5* [31], *Hand1* [40]*, Hoxb1* [41]) but lowly expresses the archetypal cardiac progenitor marker *Nkx2-5* [42] (Table 1). It also expresses more specific markers like *Mab21l2* and *Hoxd1*. Based on the expression of *Mab21l2*, we termed this region the Juxta Cardiac Field (JCF). A subsequent study by a separate group revealed a broader region of similar progenitors, earlier in development and which extended further into the extraembryonic mesoderm, as defined by *Hand1* (Fig. 1e) [23••].

The JCF has a maintained proliferative status throughout cardiac crescent development, making it distinct from the typical FHF and suggesting it could represent a maintained progenitor pool like the SHF [22••]. It also expresses a specific set of genes encoding signaling molecules involved in cardiomyocyte differentiation such as Bmp4 and Wnt2. Given the JCF’s dynamic anatomical location as well as its expression of signaling molecules, it is likely to alter local signaling environments, for example, signals from the endoderm, which can influence midline fusion of the mesodermal wings and thus potentially regulate cell fate decisions [43, 44].

Using single-cell computational approaches, it is possible to infer differentiation trajectories and investigate the molecular changes that occur during the transition from cardiac progenitor to myocyte. However, any conclusions drawn from these observations must be validated in vivo. Computational analysis of our recent single-cell RNAseq data identified two trajectories by which cardiac progenitors could generate cardiomyocytes during a developmental period spanning cardiac crescent development (Fig. 1g) [22••]. These trajectories occupied specific anatomical locations within the anterior cardiac crescent forming region of the embryo. One trajectory showed a distinct transition from SHF progenitors toward cardiomyocytes and was located in a medial and dorsal position relative to the cardiac crescent. The second was more complicated and represented the molecular convergence of both JCF and SHF progenitors, through a FHF-like state prior to cardiomyocyte differentiation. This trajectory was positioned ventral to the cardiac crescent. Lineage analysis of *Mab21l2* expressing progenitors confirmed the computational prediction that the JCF could give rise to cardiomyocytes, although we do not know what proportion of the heart this population contributes. This analysis also revealed that the JCF could contribute to the epicardium; however, we do not know whether the JCF progenitors are multipotent [23••]. Lineage analysis of the earlier *Hand1* embryonic/extraembryonic spanning domain supported this finding and showed that these earlier progenitors are multipotent. However, we did not examine early enough stages to conclude whether this FHF-like transition state was expressed prior to cardiac crescent emergence, and thus cannot conclude whether a FHF progenitor population exists that is molecularly distinct from the FHF-like transition state. Our computational analysis also suggests that SHF progenitors can give rise to cardiomyocytes via two distinct transitory cell states (FHF-like and SHF-like). This may represent a separation between the aSHF and pSHF, with the pSHF and JCF transitioning through a FHF-like state. Alternatively, it could suggest the presence of a cardiac progenitor, which can transition through different intermediate states dependent on its anatomical location, although these interpretations require further in vivo lineage exploration.

## Cardiomyocyte Lineage

Lineage represents the developmental history of a differentiated cell in the wild-type setting. Typically, this is examined by labeling populations of cells which express a given marker gene at a specific timepoint, but it is more informative when examining individually labeled cells (clonal analysis). While equated conceptually, cardiac lineages (as described by clonal analyses) and cardiac fields (molecularly defined anatomical patterns) are distinct. In the intact murine embryo, myocardial lineages were first delineated using an *nlaacZ* reporter gene in the *α-cardiac actin* locus, enabling retrospective clonal analysis [45]. Spontaneous but rare labeling events by intergenic recombination into a functional lacZ gene allow any progenitor to be uncovered, but it is retrospective. This revealed the presence of two distinct cardiac lineages termed the first and second lineage (not to be confused with the FHF and SHF) (Fig. 1h). In the looping heart at E8.5, the first lineage colonized both left and right ventricles, atrioventricular canal, and venous pole, while the second lineage contributed to the right ventricle, atrioventricular canal, venous pole, and the outflow tract. The only regions of the heart that were entirely derived from a single lineage were the left ventricle (first lineage) and outflow tract (second lineage). Rare small clones colonizing both the right and left ventricles were also identified, indicating the existence of a common cardiac progenitor, although the temporal dynamics of such a progenitor cannot be determined using this approach.

With the development of fluorescent-based clonal reporters such as confetti or mosaic analysis with double markers (MADM), a more targeted approach has been taken to assess cardiac lineages by labeling *Mesp1*-positive cardiac progenitors [46, 47]. *Mesp1* is transiently expressed in the forming mesoderm during gastrulation [48]. Using an inducible system, analysis of *Mesp1*-derived progenitors at E12.5 supported the presence of two distinct lineages but showed a greater degree of anatomical distinction compared to the earlier retrospective studies (Fig. 1h) [46]. Early mesoderm labeling resulted in clones only being detected in the left ventricle, while later mesoderm labeling resulted in clones solely in the right ventricle, atria, and outflow tract. A similar study using *Mesp1-Cre* and MADM showed that *Mesp1*-positive cardiac progenitors gave rise to distinct regions of the heart, again with no clones spanning the left and right ventricles [47]. These findings are in contrast to the earlier retrospective clonal analysis and suggest that cardiac lineage is predefined in the streak or later. This discrepancy could be related to the labeling approach used and the observation that while *Mesp1*-Cre labels the majority of the cardiac crescent, there is a population of progenitors that are not labeled. *Mesp1*-Cre has been shown to label around 70% of cardiomyocytes [49]. During heart formation, non-labeled cells are located in the medial region of the cardiac crescent (Fig. 1i). This non-labeling may reflect the transient nature of *Mesp1* expression in early progenitors (e.g., insufficient time for recombination) and the technical caveats of the labeling approaches used (e.g., temporal delay in recombination following administration to mother, uncertainty regarding the true stage of embryo labeled) or a *Mesp1*-independent differentiation pathway. The other difference between the *Mesp1-Cre-* and *nlaacZ*-based approaches is the stage of heart development examined. The *nlaacZ* approach predominantly examined looping hearts at E8.5, while the *Mesp1* studies focused on later stages once looping was complete (E10.5/E12.5) and did not image the entire heart, now possible with tissue clearing approaches such as CUBIC or Ce3D [50, 51]. Interestingly, using the inducible *Mesp1* approach clones could be detected spanning both the presumptive left and right ventricle at E8.5, raising the question as to how cardiac lineage is resolved over subsequent development.

In summary, both the *nlaacZ* and *Mesp1* clonal analysis and our computational lineage inference support the concept of two cardiomyocyte lineages; however, understanding their temporal dynamics, contribution to specific cardiac chambers, and relation to defined cardiac progenitor populations needs to be further resolved. For example, do the recently identified JCF and broader *Hand1* domain represent cells of the first lineage?

## When Is Cardiomyocyte Progenitor Fate Assigned?

To understand the fundamentals of heart formation and gain potential therapeutic insight, it is important to define when and how cardiac progenitors commit to specific cell fates (i.e., when can a cell’s fate not be reversed or transformed). During gastrulation, cardiac progenitors arise from the primitive streak at different times and in distinct anatomical locations [52–57]. For example, the earliest progenitors will emerge from a region of the primitive streak at the embryonic/extraembryonic boundary(~ E6.5). At slightly later stages of development (~ E7.0), the primitive streak elongates and cells will also ingress in a more rostral region. Given the difficulty in delineating the temporal and spatial aspects of gastrulation and the dynamic expression of genes during this short window of development, it is hard to define whether early cardiac progenitors represent predefined populations within the primitive streak or whether heart field and cardiac chamber fate is more plastic and regulated by the specific signaling environments nascent mesoderm experiences during migration. In support of an early segregation, small populations of *Mesp1*-derived cardiac progenitors expressing markers of the FHF, aSHF, and pSHF are prepatterned during early gastrulation [27]. They are thus suggested to commit to different fates and heart regions, although the clonal fate of these populations has not been directly assessed. The early specification of cardiac progenitors to distinct chamber fates has been suggested to occur within the primitive streak [58]; however, this did not assess clonal dynamics and is subject to the caveats of using recombinase-based approaches. In a human gastrulating embryo at a comparable stage of development, it was difficult to identify distinct cardiac progenitor populations, but this may represent species differences or the single embryo analyzed [59].

Contrary to the notion that cardiac progenitors represent predefined states in the primitive streak, it has been suggested that cardiac progenitor fate is governed by positional cues and the anatomical location at which they arise and add to the heart [60]. The anatomical mapping of cardiac progenitors post mesoderm migration shows that transitory differentiation states are located at the boundaries of the cardiac crescent (Fig. 1f). This highlights how morphology and cardiac cell fate decisions are inextricably linked. In support of the positional cue argument, it has been shown that early cardiac progenitors do have some plasticity and that the translocation of cardiac progenitors to different anatomical domains during early development can alter their fate [61–63]. Recent live imaging approaches have shown that during mesoderm migration, there is a considerable amount of cell-mixing [64]. The rearrangement and crossing of cell tracks highlight the potential for early cardiac progenitors to have considerable early plasticity with respect to their final cell fate. Together with the distinct location of progenitor transition states, this could suggest that patterning occurs post migration and that it is the position of cardiac progenitors within the mesodermal wings, as cardiac crescent formation and rostral folding begin that influences commitment to a certain cell fate.

In summary, while mesoderm subtypes (e.g., extraembryonic and lateral plate) arise in a spatiotemporal manner, the dynamics of mesoderm migration and the distinct location of progenitor transition states support the idea that cardiac progenitor fate isn’t rigid during early gastrulation. Understanding progenitor plasticity is of interest when examining disease and how progenitor fate is impacted when early developmental processes and gene networks are dysregulated [65]. For example, disrupting mesodermal wing migration and midline fusion results in a phenotype termed cardia bifida. This defect is characterized by the formation of two heart tubes, which independently beat and look grossly normal [66–68]; however, there is dysregulation in right and left ventricular markers [69].

## Conclusion/Outlook

Thanks to the development of single-cell resolution approaches, there has been a significant increase in our understanding and characterization of cardiac progenitors. The field is therefore in an exciting position to explore the questions raised by these recent advances. Addressing these questions will be facilitated by technological developments such as those related to high-resolution time-lapse imaging and clonal lineage analysis. With the use of light-sheet microscopy, it is now possible to image periods of mouse embryonic development spanning the onset of gastrulation to LHT formation [64, 70]. Coupling this with the ability to track single cells, as well as remove motion artifacts due to contraction, means that cardiac cell fate decisions can now be visualized during formation of the heart and thus lineages can be directly assessed. Clonal analysis techniques have also been developed which enable a more global view of lineage dynamics such as CRISPR-based genetic scars and DNA barcodes, as well as approaches which don’t require genetic manipulations, using somatic mutations or variations in mitochondrial DNA to reconstruct cell lineage [71–76]. Combining these approaches with perturbations will provide unprecedented insight into how the heart forms and identify the mechanisms and pathways regulating cardiac progenitor specification and differentiation. This insight will not only address questions of fundamental biological significance but will provide insight into the causes of congenital heart defects, a blueprint to generate biologically relevant in vitro models and aid in the development of cell-based regenerative strategies to treat disease.
